# *KRAS *analysis in colorectal carcinoma: Analytical aspects of Pyrosequencing and allele-specific PCR in clinical practice

**DOI:** 10.1186/1471-2407-10-660

**Published:** 2010-12-01

**Authors:** Magnus Sundström, Karolina Edlund, Monica Lindell, Bengt Glimelius, Helgi Birgisson, Patrick Micke, Johan Botling

**Affiliations:** 1 Molecular Pathology Unit, Department of Genetics and Pathology, Uppsala University, SE-751 85 Uppsala, Sweden; 2 Department of Oncology, Radiology and Clinical Immunology, Uppsala University, SE-751 85 Uppsala, Sweden; 3 Department of Surgery, Uppsala University, SE-751 85 Uppsala, Sweden

## Abstract

**Background:**

Epidermal growth factor receptor inhibitor therapy is now approved for treatment of metastatic colorectal carcinomas (CRC) in patients with tumors lacking *KRAS *mutations. Several procedures to detect *KRAS *mutations have been developed. However, the analytical sensitivity and specificity of these assays on routine clinical samples are not yet fully characterised.

**Methods:**

The practical aspects and clinical applicability of a *KRAS-*assay based on Pyrosequencing were evaluated in a series of 314 consecutive CRC cases submitted for diagnostic *KRAS *analysis. The performance of Pyrosequencing compared to allele-specific, real-time PCR was then explored by a direct comparison of CE-IVD-marked versions of Pyrosequencing and TheraScreen (DxS) *KRAS *assays for a consecutive subset (n = 100) of the 314 clinical CRC samples.

**Results:**

Using Pyrosequencing, 39% of the 314 CRC samples were found *KRAS*-mutated and several of the mutations (8%) were located in codon 61. To explore the analytical sensitivity of the Pyrosequencing assay, mutated patient DNA was serially diluted with wild-type patient DNA. Dilutions corresponding to 1.25-2.5% tumor cells still revealed detectable mutation signals. In clinical practice, our algorithm for *KRAS *analysis includes a reanalysis of samples with low tumor cell content (< 10%, n = 56) using an independent assay (allele-specific PCR, DxS). All mutations identified by Pyrosequencing were then confirmed and, in addition, one more mutated sample was identified in this subset of 56 samples. Finally, a direct comparison of the two technologies was done by re-analysis of a subset (n = 100) of the clinical samples using CE-IVD-marked versions of Pyrosequencing and TheraScreen *KRAS *assays in a single blinded fashion. The number of samples for which the *KRAS *codon 12/13 mutation status could be defined using the Pyrosequencing or the TheraScreen assay was 94 and 91, respectively, and both assays detected the same number of codon 12 and 13 mutations.

**Conclusions:**

*KRAS *mutation detection using Pyrosequencing was evaluated on a consecutive set of clinical CRC samples. Pyrosequencing provided sufficient analytical sensitivity and specificity to assess the mutation status in routine formalin-fixed CRC samples, even in tissues with a low tumor cell content.

## Background

Targeting of epidermal growth factor receptor (EGFR) with the monoclonal antibodies cetuximab or panitumumab prevents activation of downstream signalling molecules. Thereby cellular events such as proliferation, migration and survival are affected. Anti-EGFR therapy is recommended for patients with refractory metastatic colorectal cancer (mCRC) and is currently evaluated in clinical studies as a first line therapy in mCRC. However, activating somatic point mutations in *Kirsten RAS *(*KRAS*) are strongly associated with resistance to anti-EGFR therapy and are present in approximately 40% of colorectal tumors [1-12]. Therefore, treatment is only approved for patients harbouring a tumor with a wild-type (wt) *KRAS *gene. Consequently, robust, reliable, and sensitive methods for mutation analysis are required to stratify patients eligible for anti-EGFR therapy. Specific mutations in codon 12, 13 or 61 of the *KRAS *gene convert the gene into an active oncogene [13]. Mutations in codon 12 or 13 are the most frequent alterations in *KRAS *and represent more than 90% of all mutations. Analyses of *KRAS *in CRC clinical trials have therefore focused on these codons when relating *KRAS *mutational status to objective response or survival during EGFR inhibitor therapy. Despite being described as an activating *KRAS *mutation *in vitro*, the frequency of codon 61 mutations in human tumors is generally reported as low [2,14-18], and the clinical impact of these mutations is still under discussion [19,20].

The Pyrosequencing technology [21] has an analytical sensitivity for detection of mutations that is superior to that of Sanger (dideoxy) sequencing. Several in-house Pyrosequencing assays for detection of *KRAS *mutations in codon 12, 13 and 61 have been developed [12,14,22,23]. However, in a clinical setting there are several challenges when performing mutation analysis on DNA from routinely formalin-fixed, paraffin-embedded (FFPE) tissue samples. These include suboptimal quality of DNA due to formalin fixation, low tumor cell content in tumor tissues with abundant inflammatory cells, and insufficient starting material, e.g. minimal biopsy fragments. The practical aspects and efficiency of the CE-IVD-marked Pyrosequencing kit for analysis of *KRAS *mutations have until now not been evaluated in a clinical setting.

The TheraScreen kit (DxS Ltd, Manchester, UK) is a well-established CE-IVD-marked kit for diagnostic analysis of *KRAS *mutational status [24]. The DxS technology combines allele-specific amplification with real-time PCR for analysis of seven mutations in codons 12 and 13. With high quality DNA this method has the potential to detect ≤ 1% mutant alleles in DNA from a tumor tissue. However, this level of sensitivity is difficult to obtain in most diagnostic FFPE samples [25]. The DxS technology was used in many of the retrospective studies that evaluated the response to anti-EGFR therapy in relation to *KRAS *mutation status [4,26,27].

The aims of the present study were: (i) to describe the *KRAS *mutation spectrum in a consecutive series of CRC specimens (n = 314) referred to our laboratory for *KRAS *mutation analysis during the last two years; (ii) to evaluate the performance of Pyrosequencing compared to allele-specific PCR (DxS) on the samples (n = 56) that originated from tissues with a low tumor cell count; and (iii) to evaluate the new CE-IVD-marked versions of these techniques on a selected subset (n = 100) of the specimens.

## Methods

### Patient material

From January 2008 to June 2009, 314 consecutive FFPE CRC patient tissues were subjected to *KRAS *mutation analysis after a histological confirmation of adenocarcinoma and presence of tumor cells in haematoxylin-eosin-stained slides. In addition, the tumor cell fraction was estimated and, when possible, tissue samples were manually microdissected with a scalpel to enrich for tumor cells before DNA extraction. The project was conducted in compliance with the Helsinki Declaration. The patient samples were used in accordance with the Swedish Biobank Legislation and Ethical Review Act (approval by Uppsala Ethical Review Board, reference number: 2004:M-281 and 2009-224), including appropriate procedures for informed consent.

### DNA extraction

Depending on the size of the tissue sample, one to ten sections (10 μm thick) from the FFPE block were used for genomic DNA extraction in an EZ1 workstation using the EZ1 DNA tissue kit and EZ1 DNA paraffin section card (QIAGEN GmbH, Hilden, Germany) according to the manufacturer's instructions. The quality and concentration of the extracted DNA was determined using a NanoDrop instrument (Thermo Scientific, Wilmington, DE).

### Samples for the CE-IVD-marked PyroMark and TheraScreen assay comparison

Of the 314 samples, a subset of 100 consecutive samples was used for a direct comparison of the two CE-marked assays. Sample inclusion criteria for this part of the study were a DNA concentration of ≥10 ng/μl and a total amount of 440 ng DNA remaining after the original diagnostic *KRAS *analysis. The 100 samples were analysed for mutations in codons 12 or 13 using both methods in a single blind fashion. The sample code key was broken after all analyses were completed.

### Pyrosequencing analysis

The initial Pyrosequencing analysis of the 314 samples was performed according to the manufacturer's recommendations for PyroMark Q24 KRAS and PyroMark Q24 KRAS v2.0 assays. Briefly, 2×10 ng of genomic DNA were used for analyses of *KRAS *codons 12/13 and 61 in 2×25 μl PCR reactions. Twenty μl of each PCR product was subjected to Pyrosequencing analysis using Streptavidin Sepharose High Performance (GE Healthcare, Uppsala, Sweden), PyroMark Gold Q96 reagents, PyroMark Q24 1.0.9 software, and a Q24 instrument (QIAGEN). The mutation analysis using the CE-IVD-marked PyroMark KRAS kit (QIAGEN) in the comparative study of the 100 samples was performed according to the PyroMark KRAS Kit handbook, version 1, June 2009. Briefly, 10 ng of genomic DNA was used for the initial 25 μl PCR reaction for codon 12 and 13 mutation analysis of each sample. Ten μl of the PCR product was subjected to the Pyrosequencing reaction. The quality thresholds for the mutational analysis were a required peak height of 30 relative light units (RLU) for "passed" quality and 10 RLU for "check" quality. Samples with an initial "check" status, or with an indicated mutation signal of 2-5%, were subjected to a second round of analysis performed in triplicates. In addition, samples that failed the initial PyroMark KRAS analysis were subjected to a second round of analysis.

### Analysis using the DxS TheraScreen K-RAS Mutation Kit

The TheraScreen analysis (DxS Ltd, Manchester, UK) is an allele-specific PCR-based technology with specific primers for the seven most common *KRAS *codon 12 and 13 mutations. The assay screen for the following mutations: 12 GCT (Ala), 12 GAT (Asp), 12 CGT (Arg), 12 TGT (Cys), 12 AGT (Ser), 12 GTT (Val), and 13 GAC (Asp). Mutation analysis was performed according to instructions in the TheraScreen K-RAS Mutation Kit manual, version DU001e, January 2009 on an ABI PRISM^® ^7900HT SDS PCR instrument (Applied Biosystems Inc., Foster City, CA). In total 8×15 ng genomic DNA was used for the initial analysis of each sample. The quality thresholds when using the TheraScreen K-RAS Mutation Kit followed the recommendations in the manual, e.g. samples with a control assay with a cycle threshold (Ct) of 35 or higher should be rejected and samples with a mutation signal of Ct ≥38 should be scored as negative (wild-type).

### DNA quality control analysis

Analysis of DNA quality for samples that gave suboptimal results in both assays was performed by a multiplex PCR reaction (Specimen Control Size Ladder, InVivoScribe Technologies, San Diego, CA) for amplification of five different DNA targets (100, 200, 300, 400 and 600 basepairs), according to the manufacturer's instructions. Products were visualised by electrophoresis on a 2% agarose gel.

## Results

### Frequency and distribution of *KRAS *mutations

**Figure 1:**
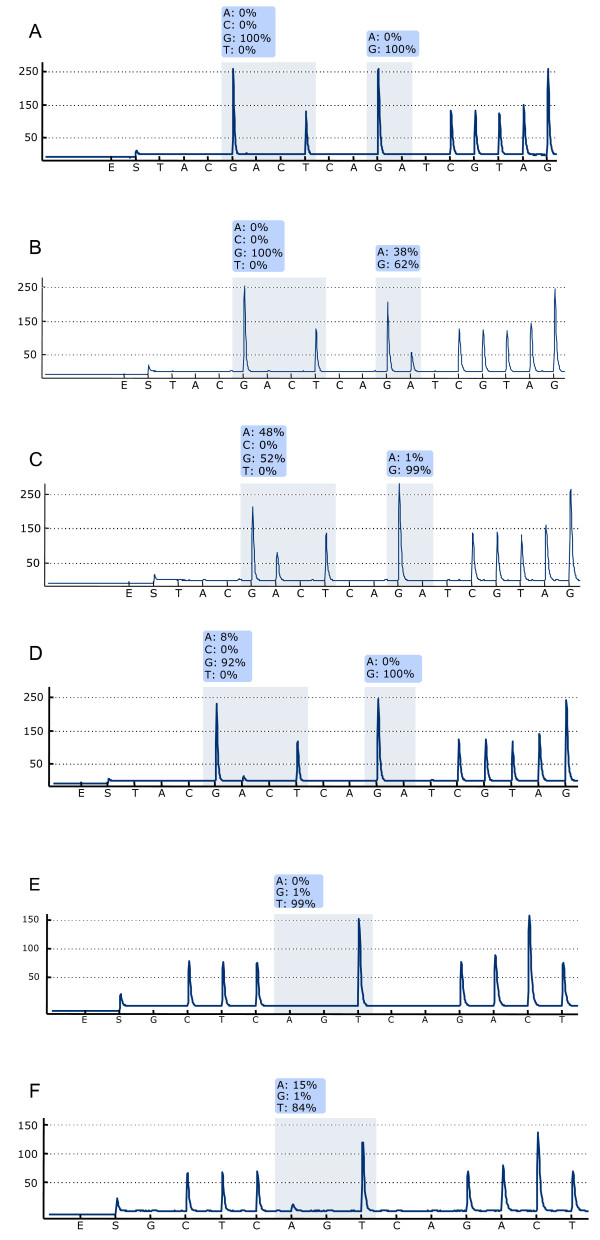
***KRAS *analysis by Pyrosequencing**. Representative examples of Pyrosequencing *KRAS *analyses of codons 12 and 13 (A-D) and codon 61 (E-F). Codon 12, 13 wt (A); codon 13 mutation GGC>GAC (B); codon 12 mutation GGT>GAT (C); codon 12 mutation GGT>GAT in DNA from tissue with less than 10% tumor cells (D); codon 61 wt (E), and codon 61 mutation (F).

During 2008 and 2009, 314 CRC samples were referred for *KRAS *mutation analysis to the Molecular Pathology laboratory at Uppsala University Hospital. By use of commercially available Pyrosequencing technologies (PyroMark Q24 KRAS and Q24 KRAS v2.0), we were able to assess the mutation status for codon 12/13 in 306 (97.5%) and codon 61 in 304 (96.8%) of the cases. See Figure 1 for representative pyrograms describing mutations in codons 12, 13 and 61.

**Figure 2:**
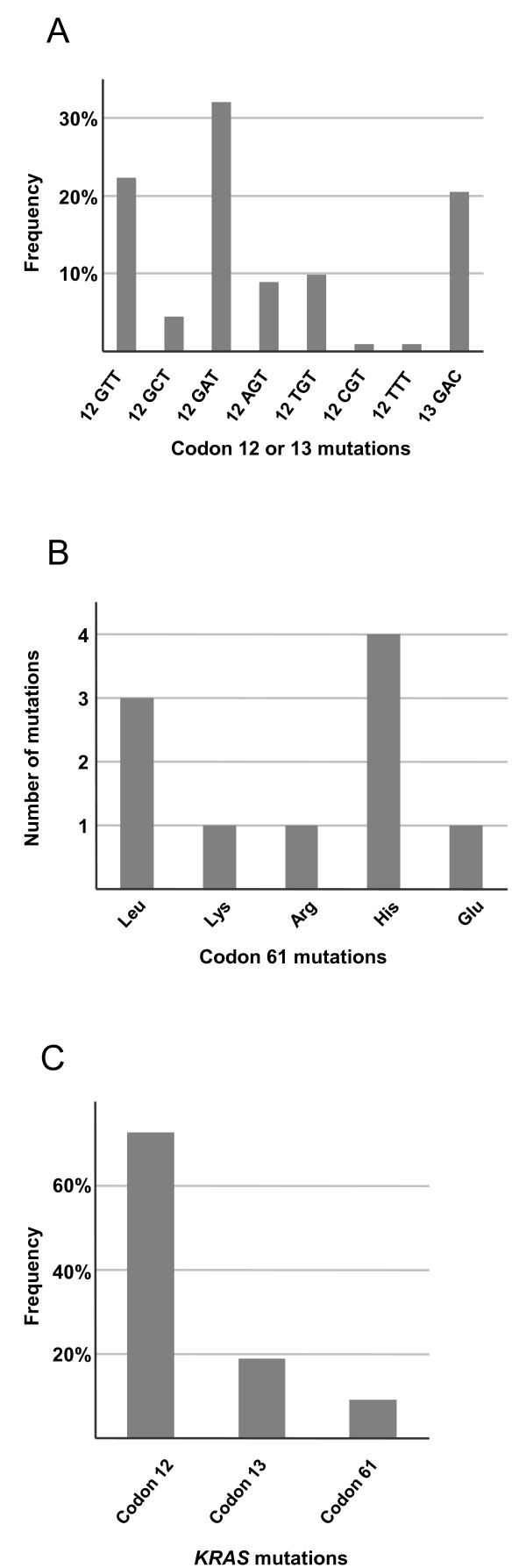
**Distributions of *KRAS *mutations**. Frequency of the detected *KRAS *codon 12 and 13 mutations (A). Distribution of mutations detected in codon 61 (B). Relative distribution of *KRAS *codon 12, 13 and 61 mutations (C).

Point mutations in codon 12/13 were identified in 112 samples (37%). In total, eight variants of codon 12/13 mutations were found (Figure 2A). Consistent with previous reports, the most common mutations were 12 GAT, 12 GTT and 13 GAC [1,2]. Beside the seven most established mutations, one unusual codon 12 mutation (GGT>TTT, Gly to Phe) was identified. Additionally, ten mutations in codon 61 were detected that were mutually exclusive with mutations in codon 12 or 13. All of these mutations lead to amino acid substitutions in the KRAS protein (Figure 2B). In conclusion, the total number of samples with *KRAS *mutations was 122 (39%) of which 10 (8.2%) were located in codon 61 (Figure 2C).

### Tumor cell content and analytical sensitivity

**Figure 3:**
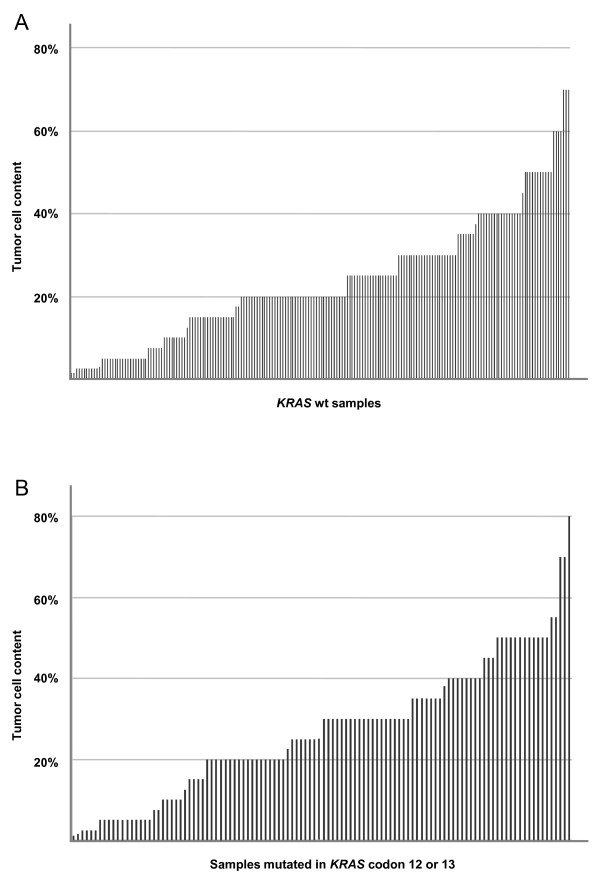
**Tumor cell content**. Estimated tumor cell content, after manual microdissection, in colorectal cancer tissues from 314 consecutive patient samples subjected to *KRAS *mutation analysis. Tumor cell content in wt samples (A) and in samples with a detected codon 12 or 13 *KRAS *mutation (B).

The high abundance of genetically normal parenchymal, stromal and inflammatory cells in human tumor tissues limits the likelihood to detect mutations in the cancer cells [28]. Therefore, tumor cell fractions were routinely estimated by a pathologist followed by manual microdissection of the tissues to enrich for tumor cells. The final mean tumor cell fraction in all analysed samples were 25% and only 11% of the samples exhibited ≥50% tumor cell content. However, no correlation was observed between tumor cell content and the ability to detect mutations by Pyrosequencing (Figure 3A, B). *KRAS *mutations were detected in many samples with a very low tumor cell count (Figure 3B), and the frequency of *KRAS *codon 12 or 13 mutations in tissues with a low (< 10%) tumor cell content (36% mutation rate) was similar to that of tissues with ≥10% tumor cells (37% mutation rate).

### Sensitivity of the Pyrosequencing mutation detection

**Figure 4:**
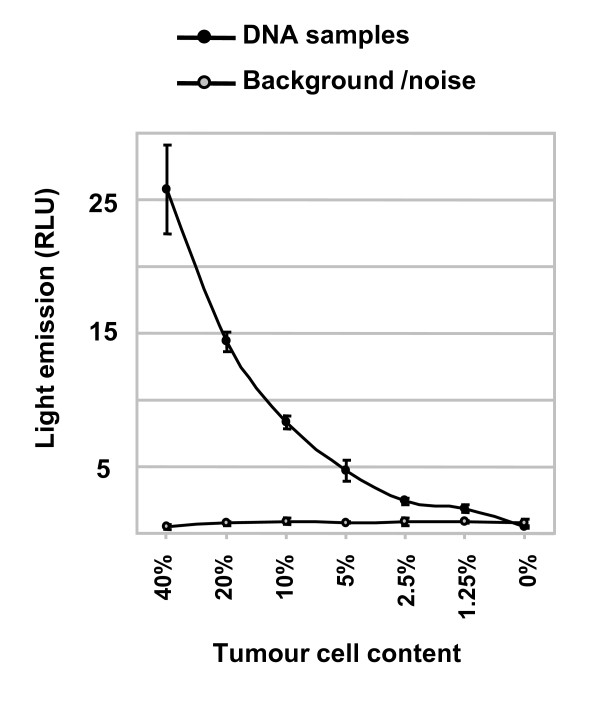
**Evaluation of the sensitivity of the Pyrosequencing technology for detection of *KRAS *mutations**. DNA from a *KRAS *codon 12 mutated tissue with a tumor cell content of 40% was serially diluted in wt DNA. Each dilution was analysed in triplicate by Pyrosequencing. The relative light units (RLU) for the mutation signal are presented.

The sensitivity of the commercially available Pyrosequencing assay to detect *KRAS *mutations in DNA extracted from FFPE tissue has not been fully evaluated. Therefore, DNA was extracted from a *KRAS*-mutated patient tissue carefully reviewed to contain 40% tumor cells, and serially diluted with wt patient DNA. The Pyrosequencing analysis of the samples show that dilutions corresponding to 1.25-2.5% tumor cells had a low, but still detectable, mutation signal (Figure 4). Similar results were obtained using the TheraScreen K-RAS Mutation kit (mutation detected down to 1.25% tumor cells) consistent with the reported sensitivity of this method (data not shown).

Our diagnostic algorithm for *KRAS *analysis includes reanalysis of all samples with < 10% tumor cells using an independent assay. Thus, samples with a low tumor cell count (n = 56) were also analysed by a mutation specific PCR method (TheraScreen K-RAS Mutation kit). All codon 12/13 mutations detected by Pyrosequencing were confirmed, and in three additional samples indications of *KRAS *mutations were found. However, of these three samples, only one met the formal analysis criteria of the TheraScreen assay, i.e. that the real-time PCR signal for a mutation had a Ct value below 38. This sample had 5% tumor cells and a codon 12 GGT>GAT mutation.

### Comparison of the CE-IVD-marked PyroMark and TheraScreen K-RAS assays

When performing clinical *KRAS *analyses, some samples will generate a borderline result due to low overall signal strength or a weak mutation signal. Validated assays, with a stringent definition of a true positive signal, are therefore essential. Since May 2009 there is a CE-marked Pyrosequencing kit (PyroMark KRAS kit, QIAGEN) available for *in vitro *diagnostic use in Europe. Therefore, we compared the performance of this new Pyrosequencing kit with the CE-marked TheraScreen K-RAS Mutation kit in a single blinded study protocol. DNA aliquots from a consecutive subset (n = 100) of the 314 CRC samples were analysed for *KRAS *mutations in codons 12 and 13 using both methods, and interpreted according to the manuals regarding procedures and criteria for positive and negative mutation results.

Eighty-nine of the 100 samples analysed with the PyroMark KRAS kit passed the quality criteria in the initial run. Four samples had an initial, and final, "failed" status (signal strength below the defined limits). Because of weak signal strength or indications of a *KRAS *mutation signal, the remaining seven samples were reanalysed in triplicates. After reanalysis, five of the samples passed the quality criteria. Thus, the mutational status of codons 12 and 13 could be defined for 94% of the samples by using this method.

With the CE-IVD-marked TheraScreen K-RAS Mutation kit, the mutational status of 89 of the 100 samples could be assessed in the initial analysis. Nine samples were rejected due to a control Ct of ≥35. The remaining two samples passed the quality criteria after reanalysis in triplicate. Thus, the total number of samples for which the mutational status could be defined using this assay was 91. Results of the failed, rejected, and reanalysed samples from both methods are outlined in Table 1.

**Table 1:** Failed, rejected, and reanalysed samples in the comparison of the PyroMark and TheraScreen K-RAS assays

DNA #	PyroMark	TheraScreen	Comments
21	ND	ND	Pyro: Failed, repeated twice. DxS: Rejected, control Ct > 35
25	WT	ND	Pyro: Check, triplicate = wt. DxS: Rejected, control Ct > 35
30	WT	ND	Pyro: Check, triplicate = wt. DxS: Rejected, control Ct > 35
37	ND	ND	Pyro: Check, triplicate = inconclusive. DxS: Rejected, control Ct > 35
54	WT	12 GTT	Pyro: Triplicate = wt. DxS: 12 GTT mutation
61	WT	ND	DxS: Rejected, control Ct > 35
64	ND	ND	Pyro: Failed. DxS: Rejected, control Ct > 35
66	12 GAT	ND	Pyro: Check, triplicate = 12 GAT mutation. DxS: Rejected, control Ct > 35
67	ND	ND	Pyro: Failed, repeated in triplicate. DxS: Rejected, control Ct > 35
89	ND	ND	Pyro: Failed. DxS: Rejected, control Ct > 35
93	ND	WT	Pyro: Check, triplicate = inconclusive. DxS: triplicate = wt
100	12 GTT	12 GTT	Pyro: Low mutation signal, triplicate = 12 GTT mutation. DxS: 12 GTT mutation

Summary of the mutational status of samples with "check", "failed" (PyroMark) or "rejected" (TheraScreen, DxS) status. ND = mutation status could not be determined.

Of the 94 samples, for which the mutational status could be defined using the PyroMark KRAS assay, 33 exhibited a *KRAS *mutation in codon 12 or 13. Similarly, 33 of the 91 passed samples in the TheraScreen mutational analysis were *KRAS*-mutated. When the DNA sample code was broken, 89 samples passed both assays and had the same mutational status. Five samples (DNA# 21, 37, 64, 67 and 89) failed the quality criteria of both assays (Table 1). Four samples (DNA# 25, 30, 61 and 66), were rejected in the TheraScreen assay, but could be defined as *KRAS *wt or codon 12 mutated (DNA# 66) when analysed by Pyrosequencing. Conversely, one sample (DNA# 93) gave inconclusive results in the Pyrosequencing assay but was scored as *KRAS *wt by the TheraScreen assay. Finally, DNA# 54 was defined as 12Val-mutated by TheraScreen analysis, but this mutation could not be unequivocally confirmed in the PyroMark assay.

### Evaluation of DNA quality in rejected samples

**Figure 5:**
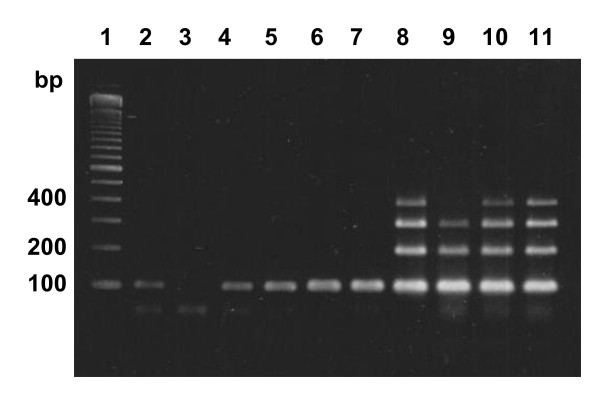
**Multiplex PCR analysis of DNA fragmentation**. DNA size ladder (lane 1). DNA samples that in the PyroMark assay: failed (lanes 2-4); had a "check" status (lanes 5-7); or "passed" (lanes 8-11).

Eight of the nine samples with a "failed" or "check" status due to low signal intensity in the PyroMark KRAS analysis were also rejected in the TheraScreen analysis due to a control Ct > 35 (Table 1). Hence, the quality of the DNA in these samples was most likely sub-optimal. To investigate why some samples gave suboptimal results, ten samples were subjected to a multiplex PCR amplification to obtain 100-600 basepair (bp) products (Figure 5). None of the six samples with a "failed" (DNA# 64, 67 and 89) or "check" (DNA# 25, 37 and 93) status in the PyroMark KRAS analysis generated an amplicon longer than 100 bp. Five of these samples (DNA# 25, 37, 64, 67 and 89) also failed the quality criteria in the TheraScreen analysis. Hence, most likely, a high degree of DNA fragmentation contributes to the failure to fulfil the quality control criteria of the assays in these samples. For the remaining four DNA samples, that passed in both assays, it was possible to amplify 300-400 bp fragments.

## Discussion

Mutated *RAS *was first identified by its ability to transform cell lines in DNA transfection experiments. It was then concluded that the transforming ability was due to activating point mutations resulting in an amino acid substitution at codon 12, 13, or 61 [13,29,30]. However, mutation analysis of codon 61, or rare mutations in codon 12 or 13, were not included in the clinical studies that lie behind the recommendation to use anti-EGFR therapies only for *KRAS *wt metastatic CRC [31,32].

In our cohort of 314 colorectal cancer patients, 39% (n = 122) of the samples were mutated in *KRAS *codon 12, 13 or 61. As expected, most mutations were located in codon 12 or 13 (n = 112) and represented well-established types of mutations in CRC [1]. One unusual codon 12 mutation (GGT > TTT) was also identified. This mutation has, to our knowledge, only been reported three times [2,33-35]. The frequency of codon 61 mutations is generally believed to be low. However, similar to a recent study [19], 8% (n = 10) of the detected *KRAS *mutations in our cohort were located in codon 61. Four of these result in a change of the wt glutamic acid to histidine. In the infrequent, but highly malignant, signet ring cell CRC *KRAS *codon 12 and 13 mutations are less frequent than in other CRC subtypes [36]. Interestingly, more codon 61 histidine substitutions than codon 12 or 13 mutations have been reported in signet ring cell CRC [37]. Although the distribution and relative frequencies of the mutation types can vary between different populations of CRC patients (reviewed by Packham *et al. *[12]), we argue that codon 61 mutations are more common than generally believed. The reason for the low incidence of codon 61 mutations reported in the Catalogue of somatic mutations in cancer database (0.3%) [2] and elsewhere (0-1.6%) [14-18] might be due to the use of sub-optimal sequencing methods or, simply, the widespread use of methods limited to codon 12 and 13 analysis. The clinical implication of *KRAS *codon 61 mutations in the treatment of CRC patients with EGFR inhibitors is not fully understood. However, a recent study indicates that codon 61 mutations are associated with resistance to cetuximab treatment [20].

The analytical sensitivity and specificity to detect a *KRAS *mutation in a clinical CRC sample will depend on (i) the technical ability to detect mutated alleles in a background of normal wt alleles, (ii) the sensitivity to DNA degradation in terms of DNA template target lengths, and (iii) the defined thresholds and signal strengths required for a positive or negative mutation call. The presence of normal cells, such as infiltrating inflammatory cells and stromal cells, will reduce the possibility to detect cancer-associated somatic mutations in a tumor sample. In clinical practice, even after manual microdissection, samples with a low tumor cell fraction are relatively common. In our series 18% of the samples had a final tumor cell content of < 10%. For these samples direct Sanger sequencing will not be sensitive enough as a tumor cell fraction of at least 25-30% is believed to be required for mutation detection by this method [25]. Hence, a more sensitive method is needed for an accurate analysis of these samples. Previous studies using either in-house developed, or PyroMark v2.0, Pyrosequencing assays have defined a detection limit of 5% mutant alleles [22,38]. According to the TheraScreen (DxS) manual, this kit has a reported sensitivity of 1% (of mutated alleles among wt alleles). This would correspond to a tumor cell content of 2% when detecting a heterozygote mutation in the tumor cells. Here we report that a similar sensitivity can be reached using the Pyrosequencing kit. We believe that the titration experiment performed in this study, using tumor and normal DNA from FFPE tissue, possibly better reflects the true diagnostic situation compared to dilution experiments with recombinant DNA or mixtures of non-fixed cultured cell lines. However, we find that both assays are sensitive to poor DNA quality. Indeed, DNA fragmentation induced by formalin fixation and/or degradation due to necrosis will reduce the success rate of PCR amplification, and might potentially introduce mutation artefacts [39,40].

In a blinded sub-study of 100 CRC samples, we compared the two CE-marked mutation detection kits, PyroMark KRAS and TheraScreen K-RAS mutation kit, by adhering to the manufacturers' protocols and guidelines for interpretation of the results. The mutational status could be defined for 94 and 91 samples, respectively, and both assays detected the same number of mutated samples. After analysis of DNA fragmentation, we concluded that suboptimal DNA quality was the reason that some samples did not pass the quality thresholds. Analysis of such samples resulted in a high control Ct (TheraScreen) or low signal strength (PyroMark). Both assays uncovered identical mutations in 32 of the samples. In addition, one sample that was rejected in the TheraScreen assay was found *KRAS *mutated when analysed with the PyroMark assay. Conversely, a mutation defined by the TheraScreen assay in one sample could not be exclusively confirmed by the PyroMark assay. Our experience when comparing the two assays in clinical practice is that the TheraScreen assay is possibly slightly more sensitive in terms of detecting low numbers of mutated alleles. However, as this assay is dependent on several real-time PCR reactions in combination with a strict regulatory definition of true mutations and thresholds for rejection (in terms of Ct values), this assay seems to be more dependent on high DNA quality in comparison to the PyroMark assay. Moreover, less DNA is needed for the PyroMark assay (10 ng) in comparison with the TheraScreen method (8×2-20 ng). The amount of DNA required for a mutation analysis can be of importance when analysing minimal colonoscopy fragments or core needle biopsies. Similar to our DxS TheraScreen results, a failure rate of 5-10% have been reported when analysing CRC samples for *KRAS *mutations using the DxS technology. The failed samples either had limited material or poor quality of the DNA [41,42]. In comparison, when analysed with Pyrosequencing, the failure rate was 2.5% for the 314 consecutive CRC samples in this study. Finally, we want to emphasise that the criteria for "mutations" or "rejections", as defined by manufacturers of kits, or individual molecular pathology laboratories, adds a subjective dimension to the interpretation of the results that goes beyond a pure technical comparison of different assays.

## Conclusions

Our conclusion from the *KRAS *analysis of the 314 clinical samples together with data from the comparison of the two CE-marked assays is that both assays are well suited for *KRAS *mutation analysis of DNA from FFPE CRC samples. Both assays have a sufficient analytical sensitivity to efficiently detect *KRAS *mutations even in samples with < 10% tumor cells.

## Competing interests

Dr. Glimelius reports receiving financial support from Merck Serono for an earlier clinical trial of CRC treatment. No other potential conflict of interest relevant to this article was reported.

## Authors' contributions

MS, KE and ML carried out the mutation analyses and interpreted the mutation data. PM and JB performed the pathological examination of the tissue sections and helped to interpret the mutation data. MS, PM and JB designed the study and drafted the manuscript. KE, BG and HB participated in the design of the study and helped to draft the manuscript. All authors have given final approval of the version to be published.

## Pre-publication history

The pre-publication history for this paper can be accessed here:

http://www.biomedcentral.com/1471-2407/10/660/prepub
